# Elevated Levels of Lead (Pb) Identified in Georgian Spices

**DOI:** 10.5334/aogh.3044

**Published:** 2020-09-28

**Authors:** Bret Ericson, Levan Gabelaia, John Keith, Tamar Kashibadze, Nana Beraia, Lela Sturua, Ziad Kazzi

**Affiliations:** 1 Pure Earth, New York, NY, US; 2 National Center for Disease Control and Public Health, Tbilisi, GE; 3 Petre Shotadze Tbilisi Medical Academy, Tbilisi, GE; 4 Emory University, Atlanta, GA, US

## Abstract

**Background:**

Human lead (Pb) exposure can result in a number of adverse health outcomes, particularly in children.

**Objective:**

An assessment of lead exposure sources was carried out in the Republic of Georgia following a nationally representative survey that found elevated blood lead levels (BLLs) in children.

**Methods:**

A range of environmental media were assessed in 25 homes and four bazaars spanning five regions. In total, 682 portable X-Ray Fluorescence measurements were taken, including those from cookware (n = 53); paint (n = 207); soil (n = 91); spices (n = 128); toys (n = 78); and other media (n = 125). In addition, 61 dust wipes and 15 water samples were collected and analyzed.

**Findings:**

Exceptionally high lead concentrations were identified in multiple spices. Median lead concentrations in six elevated spices ranged from 4–2,418 times acceptable levels. Median lead concentrations of all other media were within internationally accepted guidelines. The issue appeared to be regional in nature, with western Georgia being the most highly affected. Homes located in Adjara and Guria were 14 times more likely to have lead-adulterated spices than homes in other regions.

**Conclusions:**

Further study is required to determine the source of lead contamination in spices. Policy changes are recommended to mitigate potential health impacts. The results of this study contribute to a growing body of evidence that points to adulterated spices as a significant source of human lead exposure.

## Background and Objectives

The Georgian National Center for Disease Control and Public Health (NCDC) and United Nations Children’s Fund (UNICEF) completed a Multiple Indicator Cluster Survey (MICS) from September to December 2018 that included a nationally representative assessment of 1,578 children’s blood lead levels (BLLs). The study found that 41% of children (age 2–7 years) had BLLs equal to or exceeding the U.S. Centers for Disease Control’s reference level of 5 µg/dL and that16% of the 1,578 children had BLLs exceeding 10 µg/dL [1,2,3]. Low-level lead exposure can result in a number of adverse health outcomes in the neurological and cardiovascular systems [4,5,6]. To investigate potential sources of lead exposure present in the homes of assessed children a team comprised of staff from the NCDC and the nongovernmental organization Pure Earth (NY, USA) conducted site visits to 25 Georgian homes and four bazaars in July 2019 assessing a range of media for lead content including soil, dust, paint, water, spices, toys, and cookware.

## Methods

Sixteen of the 25 homes were selected on the basis of having a child with a BLL > 30 µg/dL, while nine were selected on the basis of having a child with a BLL < 5 µg/dL. The mean BLL in the < 5 µg/dL group was 2.81 µg/dL (range: 1.6–4.01) while the mean BLL in the > 30 µg/dL group was 35.43 µg/dL (range: 32.29–38.57). The mean age for children in the comparison group was 4.2 years (range: 2.7–5.7), while that for the elevated group was 5.1 years (range: 4.1–6.1). The sex of the children was not recorded.

The homes were located in the following five regions: Adjara (n = 9); Guria (n = 5); Imereti (n = 7); Shida Kartli (n = 3); and Tbilisi (n = 1). Spices were procured in bulk and assessed from bazaars in Adjara (n = 2), Imereti (n = 1), and Tbilisi (n = 1). Bazaar assessments were not initially contemplated in the study design and were only conducted after spices were found to be adulterated with lead during home assessments. The bazaars were selected based on logistical convenience. As a result, no bazaars were assessed in Guria or Shida Karti.

The study relied heavily on field-portable instrumentation confirmed in part by laboratory wet techniques. In total, 682 portable X-Ray Fluorescence (pXRF) measurements were taken, including those from cookware (n = 53); paint (n = 207); soil (91); spices (n = 128); toys (n = 78); and ‘other’ (n = 125) a category comprised of a range of media including cosmetics, unpainted furniture, and foodstuffs. All pXRF measurements were taken *in situ* with a ThermoFisher Scientific Niton XL3T 700 Alloy Analyzer with a 50kV Au Anode X-Ray Tube (Waltham, Massachusetts, USA). Media assessed in homes were selected on the basis of a literature review and expert consultation. In the case of bazaars, only spices were investigated.

Spice samples of approximately 100 grams were collected and stored in individually labeled Nasco Whirl-Pak® polyethylene bags. Each sample was placed on an available surface such as a floor or table and analyzed with the pXRF in soil mode. Prior to analysis, the surface was measured for detectable lead with the pXRF and confirmed to be less than the lower limit of detection (LOD) (i.e. < 8 mg/kg). A smaller subset of samples (n = 15) were couriered to EMSL Analytical, Inc. (Cinaminson, NJ, USA) for analysis with method AOAC 2013.06 using Inductively Coupled Mass Spectrometry (ICP-MS) [7].

All available spices in a household were analyzed with the pXRF. In two cases, ‘spice’ samples included table salt assessed at the homeowner’s request. The type of spice was recorded for only 65 of the 128 samples. This was due in part to poor or nonexistent labelling and a methodological gap in earlier assessments that did not account for recording spice type. Of the 128 spice samples, 98 were collected from homes and 30 were collected at bazaars in Adjara (n = 17), Imereti (n =7), and Tbilisi (n = 6).

In addition to pXRF measurements, 61 dust wipes were collected in a manner consistent with ASTM E 1728–16 and analyzed at a certified laboratory in the US (EMSL Analytical, Inc., Cinaminson, NJ, USA) using Flame Atomic Absorption Spectrometry (AAS) with a lower detection reporting limit of 10 µg/wipe (Cinaminson, NJ, USA) (ASTM, 2016). Fifteen tap water convenience samples (250 ml) were analyzed with a Varian® (Palo Alto, CA, USA) inductively coupled atomic emission spectrometer (ICP-AES) at a private nationally certified laboratory (Multitest, Inc, Tbilisi, Georgia) using method GOST 31866-2012 (Russia) as well as with an Agilent® (Santa Clara, CA, USA) inductively coupled optical emission spectrometer (ICP-OES) by the Georgian Ministry of Agriculture. Finally, all cookware and at least one paint sample in each home were assessed with 3M LeadCheck® swabs (Maplewood, MN, USA). LeadChecks® are colorimetric qualitative field tests for leachable lead in paint and other surfaces. When utilized on a surface containing > 1 mg/cm^2^ Pb, LeadChecks® turn visibly and distinctively red, having a false positive rate of < 1 % (Battelle, 2012). LeadChecks® have also been used as a screening tool for leachable lead in cookware at levels < 3 µg [8,9].

The following regulatory standards were used to guide the investigation: US Environmental Protection Agency (USEPA) drinking water action level of 15 ppb [10]; USEPA residential dust action levels of 40 µg/ft^2^ for floors and 250 µg/ft^2^ for windows [11]; USEPA action level for bare soil where children play of 400 mg/kg [11]; US Consumer Protection and Safety Commission toys action level of 100 mg/kg [12]; the New York City Department of Health and Mental Hygiene spices reference level of 2 mg/kg [13]; and the Mexican Secretary of Health action level of 2 µg/ml leachable lead in ceramic cookware [14].

Data analysis, which included logistic regression and the generation of descriptive statistics, was conducted with Stata 15.1 statistical software [15]. To account for LOD bias in pXRF measurements, the LOD was divided by the square root of two and replaced with the result [16]. In the case of spices, pXRF measurements were adjusted to conform to ICP-MS results.

## Results

Spice Pb measurements revealed highly elevated concentrations. Median lead concentrations of all other media were found to be within internationally accepted levels, with a limited number of exceedances. Three of 78 toys exceeded the applied standard; four of 61 dust wipes exceeded the applied standard.

The following six commonly used spices had elevated lead concentrations: coriander (median: 8 mg/kg; IQR: 4–37); khmeli suneli kharcho (median: 742 mg/kg; IQR: 4–4,218); kviteli kvavili (median: 4,837 mg/kg; IQR: 4–6,727); svanuri marili (median: 1,346 mg/kg; IQR: 838–1,646); turmeric (median: 1,897; IQR: 1,181–2,612); and utsko suneli (median: 13 mg/kg; IQR: 6.4–21). The median concentration for these six spices exceeded the reference levels applied in this study by 4x, 371x, 2,418x, 673x, 949x, and 6x, respectively [13]. The highest spice Pb concentration identified was 20,058 mg/kg (kviteli kvavili). Tables 1, 2, and 3 present the main findings.

**Table 1:** Adjusted lead (Pb) concentrations in spices (mg/kg) with at least two measurements.

Spice	n	Low	25 % IQR	Median	75 % IQR	High

Homes (n = 25)						
**black pepper**	5	< LOD	< LOD	< LOD	< LOD	48.43
**coriander**	5	< LOD	4.14	8.43	11.45	33.37
**khmeli suneli kharcho**	7	< LOD	13.62	741.98	4,218.21	4,363.98
**kviteli kvavili (‘yellow flower’/marigold mixture)**	7	< LOD	< LOD	4,837.22	18,293.09	20,058.61
**qondari**	3	< LOD	< LOD	< LOD	< LOD	50.23
**red pepper**	7	< LOD	< LOD	< LOD	< LOD	< LOD
**svanuri marili**	6	< LOD	838.11	1,345.53	1,645.53	2,276.74
**table salt**	2	< LOD	< LOD	6.02	7.89	7.89
**turmeric**	2	466.39	466.39	1,897.06	3,327.72	3,327.72
**utsko suneli (blue fenugreek mixture**	6	< LOD	6.42	13.12	21.23	1,075.84
**spice (undefined)**	45	< LOD	< LOD	< LOD	5.2	1,069.81
**Bazaars (n = 4)**						
**khmeli suneli kharcho**	4	< LOD	< LOD	1,969.86	4,200.31	4,465.06
**kviteli kvavili (‘yellow flower’/marigold mixture)**	6	< LOD	< LOD	5,273.82	6,727.14	10,564.79
**spice (undefined)**	20	< LOD	< LOD	< LOD	87.14737	846.12

* Single samples of bazhe suneli, carraway and zaprana were taken but are not included in the above.

**Table 2:** Lead concentrations in various media, method of analysis with lower limits of detection, and applicable regulatory standard.

Medium	Analysis method	n	Unit	Median	IQR	Range	LOD	Regulatory value applied

**Cookware**	pXRF	53	per cent	0.011	0.011–0.086	0.00056–3.395	0.00042%	*n/a*
**Cookware**	LeadCheck®	53	positive or negative	neg.	neg.	neg.	1 mg/cm^2^ or 3 ug/ml	2 ug/ml
**Drinking water**	Varian® ICP-AES with GOST 31866-2012 (Russia)	15	ppb	0.8	0.25–1.6	0.067–7.56	undefined	15
**Dust (floors)**	FAAS CPGM 7303.019B	37	µg/ft^2^	< LOD	< LOD	< LOD–240	65.76	40
**Dust (windows/elevated surfaces)**	FAAS CPGM 7303.019B	24	µg/ft^2^	< LOD	< LOD	< LOD–600	65.76	250
**Paint**	pXRF	207	mg/cm^2^	< LOD	< LOD –0.06	< LOD–0.95	0.0141	1 mg/cm^2^
**Soil**	pXRF	91	ppm	16.1	10.53–27.51	5.65–842.7	4.14	400 ppm
**Spices**	pXRF	128	mg/kg	< LOD	< LOD–110.23	< LOD–20,058.61	4.14	2 mg/kg
**Toys**	pXRF	78	ppm	< LOD	< LOD	< LOD –1,098.37	38.89	100 ppm

**Table 3:** Spice Pb concentrations and the number of children with BLLs > 5 ug/dL in 2018 MICs study by region.

Region	n	% of children > 5 ug/dL^3^	Low	25 % IQR	median	75 % IQR	High

**Adjara**	73	85.4	< LOD	< LOD	11.44	846.12	20,058.61
**Guria**	16	73.2	< LOD	< LOD	7.56	140.89	3,461.71
**Imereti**	25	60.8	< LOD	< LOD	< LOD	< LOD	3,327.72
**Shida Kartli**	2	21.4	< LOD	< LOD	< LOD	< LOD	< LOD
**Tbilisi**	12	30.1	< LOD	< LOD	< LOD	< LOD	22.15

Of the 98 spice samples collected within homes, 42 (43 %) had detectable Pb concentrations. The median spice Pb concentration of this elevated group was 88 mg/kg (IQR: 14–1,645).

**Figure 1:**
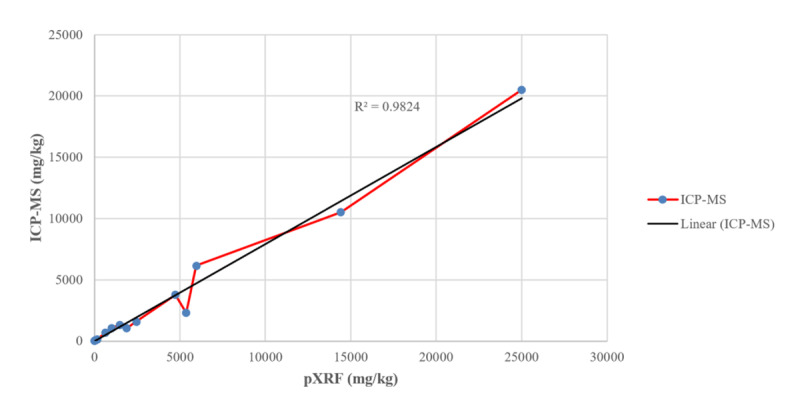
Raw (non adjusted) pXRF and ICP-MS measurements of lead in 15 spices.

**Figure 2:**
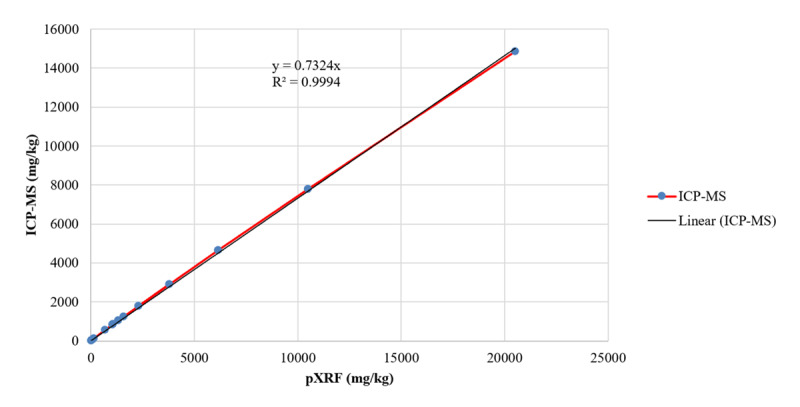
Adjusted pXRF and ICP-MS measurements of lead in 15 spices.

The pXRF performed well against ICP-MS spice Pb measurements with an R^2^ of 0.9824, indicating that the pXRF may be an adequately robust field-based assessment instrument for spices. Assuming a linear relationship, ICP-MS spice Pb measurements were approximately 73% of pXRF measurements. All pXRF spice Pb measurements were adjusted accordingly. A comparison of pXRF and ICP-MS measurements is presented in Figures 1 and 2.

Homes located in Adjara or Guria (in western Georgia) were 14 times (95%CI: 1.9–105; p = 0.01) more likely to have at least one spice exceeding reference levels. Of the 17 spices procured at bazaars in Adjara, 14 (82 %) had detectable lead concentrations. The median lead concentration for all 17 samples procured in Adjara bazaars was 233 mg/kg (IQR: 22–4,465). No spices procured in either Tbilisi or Imereti bazaars were found to have detectable lead concentrations. The data seemed to indicate that children living in households with at least one elevated spice concentration or living in Adjara or Guria were more likely to have elevated BLLs though a statistically significant relationship could not be identified, likely owing in part to the small sample size of only 25 homes.

## Discussion

The results are generally consistent with other studies, which have identified exceptionally high lead levels in Georgian spices [13,17]. Hore, *et al* (2019) for example, found a maximum concentration of 48,000 mg/kg Pb in kviteli kvavili used in New York City and carried in from Georgia.

Results from the analysis of other media are broadly consistent with studies carried out elsewhere. Lead-based paint, which is not legislatively banned in Georgia, remains widely available in markets around the world with only 38% of countries having such bans in place [18]. However despite its availability, there is a marked paucity of recent studies identifying lead-based based as a significant source of environmental lead exposure [19]. Soil lead concentrations were exceptionally low approaching near background levels [20,21]. This may have been due in part to the rural setting of many of the assessed homes and subsequent low levels of automobile traffic. There is also an ostensible absence of industrial sources of lead contamination.

Lead-adulterated spices seem most pronounced in the Adjara region in the western part of the country, where the MICS study found that 85% of children had BLLs exceeding 5 µg/dL [1]. These results contribute to a growing body of evidence that points to adulterated spices as a significant source of human lead exposure [22,23,24]. Lead is likely added to Georgian spices to enhance color. In their investigation of Bangladeshi turmeric, Forsyth, et. al. (2019), for instance, identified the use of lead chromate (PbCrO_4_) as a yellow pigment [22]. Given the minimal increase in weight at the concentrations identified it would seem unlikely that the addition of lead is for this purpose, as has been indicated elsewhere [25]. Further investigation is required to identify contamination sources and could include improved speciation of samples. The results of this study indicate that pXRF instrumentation may be a useful screening tool to help ensure food safety.

This study has a number of limitations. Among them the small sample size of 25 homes and four bazaars limits the extent to which the results can be generalized for Georgia as a whole. In addition, the heavy reliance on field-based instrumentation may adversely impact the accuracy of some of the measurements. Finally, the LOD of some analytical methods actually exceeded the regulatory standard applied. Each of these limitations are, however, somewhat muted by the exceptionally high levels of spice Pb levels and the significant health risk they pose.

## Conclusion

Lead-adulterated spices pose a significant and credible public health risk to Georgians. Additional investigation is required to effectively characterize the cause of the contamination and develop effective policy. Monitoring, regulatory action, and strategic risk communication are required to mitigate adverse health impacts and address public health concerns. Portable XRF instrumentation appears to be a robust tool for the field assessment of spice Pb concentration and could form a component of such a response. The results of this study contribute to a growing body of evidence that point to lead-adulterated spices as a significant source of exposure.

## References

[1] Kazzi Z, Gabelaia L, Shengelia L, et al. Lessons learned through the journey of a medical toxicologist while characterizing lead hazards in the Republic of Georgia. J Med Toxicol. 2019: 9–11. DOI: 10.1007/s13181-019-00744-9 PMC6942075 31728957

[2] Centers for Disease Control and Prevention. CDC – Lead – Home Page 2017 https://www.cdc.gov/nceh/lead/. Accessed May 26, 2017.

[3] National Statistics Office of Georgia. 2018 Georgia Multiple Indicator Cluster Survey, Survey Report; 2019.

[4] Lanphear BP, Rauch S, Auinger P, Allen RW, Hornung RW. Low-level lead exposure and mortality in US adults: A population-based cohort study. Lancet Public Heal. 2018; 3(4): e177–e184. DOI: 10.1016/S2468-2667(18)30025-2 29544878

[5] Budtz-Jørgensen E, Bellinger D, Lanphear B, et al. An international pooled analysis for obtaining a benchmark dose for environmental lead exposure in children. Risk Anal. 2013; 33(3): 450–461. DOI: 10.1111/j.1539-6924.2012.01882.x 22924487

[6] ATSDR. ATSDR – Toxicological Profile: Lead 2007 https://www.atsdr.cdc.gov/toxprofiles/tp.asp?id=96&tid=22. Accessed November 10, 2016.

[7] Julshamn K, Maage A, Norli HS, et al. Determination of arsenic, cadmium, mercury, and lead in foods by pressure digestion and inductively coupled plasma/mass spectrometry: First action 2013.06. J AOAC Int. 2013; 96(5): 1101–1102. http://www.ncbi.nlm.nih.gov/pubmed/24282954 Accessed August 24, 2019. DOI: 10.5740/jaoacint.13-143 24282954

[8] Sheets RW. Use of home test kits for detection of lead and cadmium in ceramic dinnerware. Sci Total Environ. 1998; 219(1): 13–19. DOI: 10.1016/S0048-9697(98)00233-2 9770321

[9] Valles-Medina AM, Osuna-Leal AI, Martinez-Cervantes ME, Castillo-Fregoso MC, Vazquez-Erlbeck M, Rodriguez-Lainz A. Do glazed ceramic pots in a Mexico-US border city still contain lead? 2014 DOI: 10.1155/2014/474176 PMC4897465 27379279

[10] US EPA O. Lead and Copper Rule 2019 https://www.epa.gov/dwreginfo/lead-and-copper-rule. Accessed August 29, 2019.

[11] USEPA. Lead; Identification of dangerous levels of lead. 1998; 63 https://www.gpo.gov/fdsys/pkg/FR-1998-06-03/pdf/98-14736.pdf. Accessed September 26, 2017.

[12] CPSC. Total Lead Content Business Guidance; Small Entity Compliance Guide | CPSC.gov 2019. https://www.cpsc.gov/Business--Manufacturing/Business-Education/Lead/Total-Lead-Content-Business-Guidance-and-Small-Entity-Compliance-Guide. Accessed August 29, 2019.

[13] Hore P, Alex-oni K, Sedlar S, Nagin D. A spoonful of lead : A 10-year look at spices as a potential source of lead exposure. Public Heal Manag Pract. 2019; 25(1): 63–70. DOI: 10.1097/PHH.0000000000000876 30507772

[14] Secretaria de Salud M. NORMA Oficial Mexicana NOM-231-SSA1-2002.

[15] StataCorp. LP. Stata Statistical Software: Release 15. Published online 2017 https://www.stata.com.

[16] CDC. NHANES Environmental Chemical Data Tutorial – Important Analytic Considerations and Limitations – Task 2. https://www.cdc.gov/nchs/tutorials/environmental/critical_issues/limitations/Info2.htm. Accessed July 11, 2020.

[17] Woolf AD, Woolf NT. Childhood lead poisoning in 2 families associated with spices used in food preparation; 2005 DOI: 10.1542/peds.2004-2884 16061585

[18] UNEP. Update on the Global Status of Legal Limits on Lead in Paint; 2019.

[19] Ericson B, Hariojati N, Caravanos J, Susilorini B, Crampe L, Taylor MP. Lead Based Paint Exposure Risk in Jakarta, Indonesia. International Symposisum of Environmental Epidimiology; 2017 DOI: 10.1289/isee.2017.2017-972

[20] Alloway BJ, (ed.) Heavy Metals in Soils Vol 22 Netherlands: Springer; 2013 DOI: 10.1007/978-94-007-4470-7

[21] Smith DB, Cannon WF, Woodruff LG, Solano F, Kilburn JE, Fey D. Geochemical and Mineralogical Data for Soils of the Conterminous United States. U.S. Geological Survey Data Series 801; 2013 https://pubs.usgs.gov/ds/801/ DOI: 10.3133/ds801

[22] Forsyth JE, Nurunnahar S, Islam SS, et al. Turmeric means “yellow” in Bengali: Lead chromate pigments added to turmeric threaten public health across Bangladesh. Environ Res. 2019; 179: 108722 DOI: 10.1016/j.envres.2019.108722 31550596

[23] Cowell W, Ireland T, Vorhees D, Heiger-Bernays W. Ground turmeric as a source of lead exposure in the United States. Public Health Rep. 2017; 132(3): 289–293. DOI: 10.1177/0033354917700109 28358991 PMC5415259

[24] Angelon-Gaetz KA, Klaus C, Chaudhry EA, Bean DK. Lead in spices, herbal remedies, and ceremonial powders sampled from home investigations for children with elevated blood lead levels—North Carolina, 2011–2018. MMWR Morb Mortal Wkly Rep. 2018; 67(46): 1290–1294. DOI: 10.15585/mmwr.mm6746a2 30462630 PMC6289082

[25] Angelon-Gaetz KA, Klaus C, Chaudhry EA, Bean DK. Lead in spices, herbal remedies, and ceremonial powders sampled from home investigations for children with elevated blood lead levels—North Carolina, 2011–2018. MMWR Morb Mortal Wkly Rep. 2018; 67(46): 1290–1294. DOI: 10.15585/mmwr.mm6746a2 30462630 PMC6289082

